# Supplementary figures and images for: Phytophthora zoospores display klinokinetic behaviour in response to a chemoattractant
Source: PLoS Pathog. 2024 Sep 30;20(9):e1012577. doi: 10.1371/journal.ppat.1012577 (PMC11554144; doi:10.1371/journal.ppat.1012577)

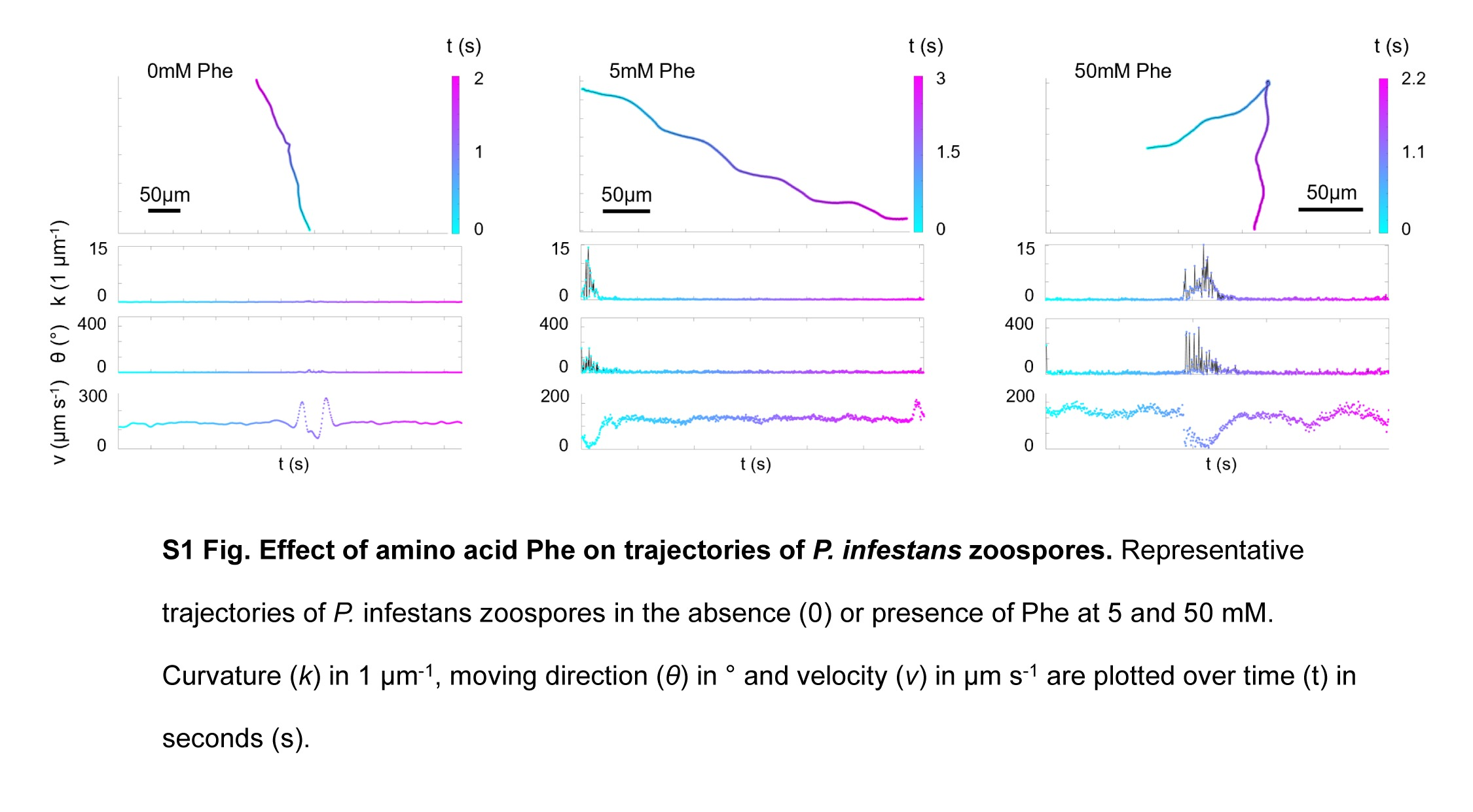

Supplement: S1 Fig — Representative trajectories of P. infestans zoospores in the absence (0) or presence of Phe at 5 and 50 mM. Curvature (k) in 1 μm-1, moving direction (θ) in ° and velocity (v) in μm s-1 are plotted over time (t) in seconds (s). (TIF) [file ppat.1012577.s001.tif]

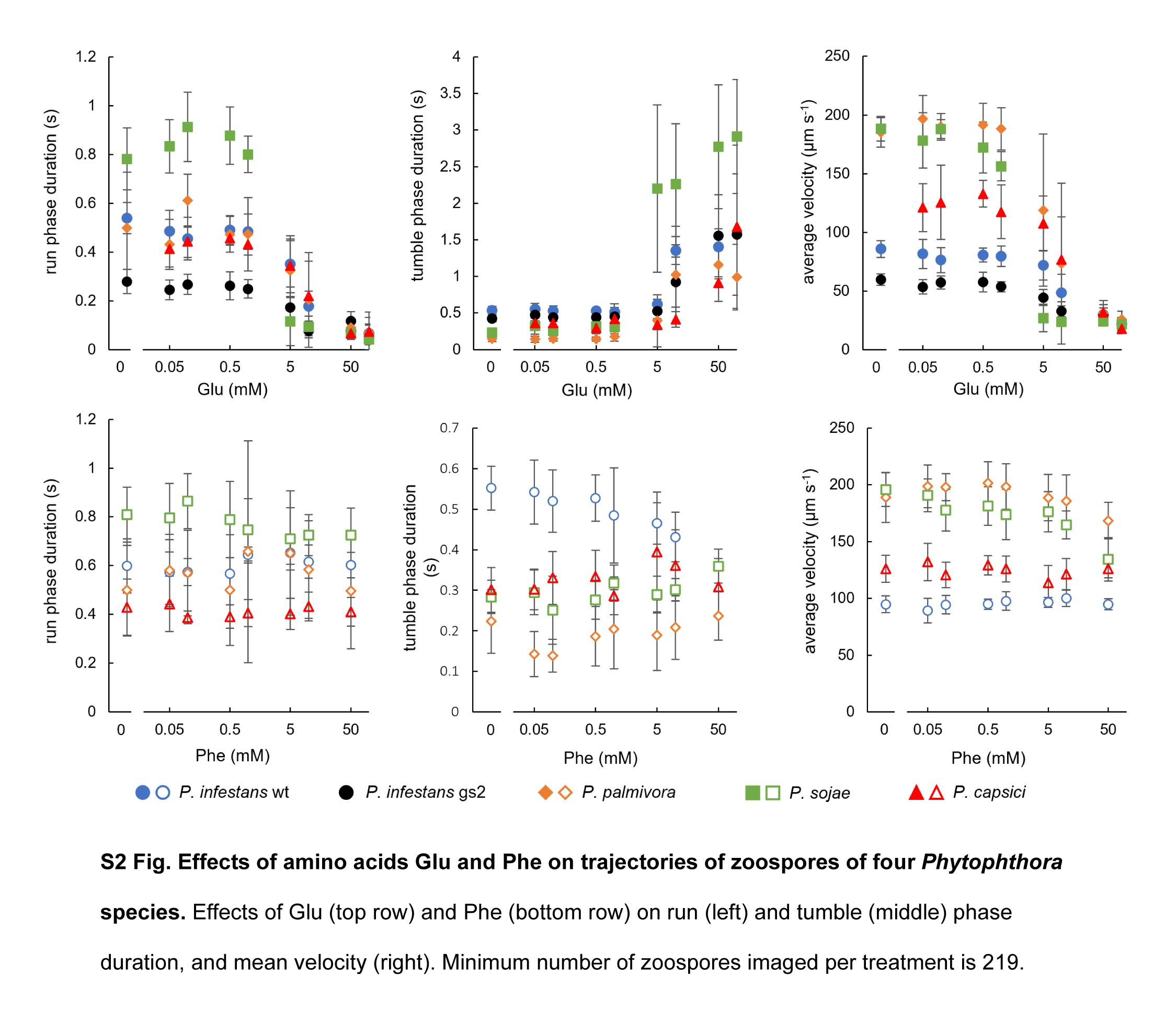

Supplement: S2 Fig — Effects of Glu (top row) and Phe (bottom row) on run (left) and tumble (middle) phase duration, and mean velocity (right). Minimum number of zoospores imaged per treatment is 219. (TIF) [file ppat.1012577.s002.tif]

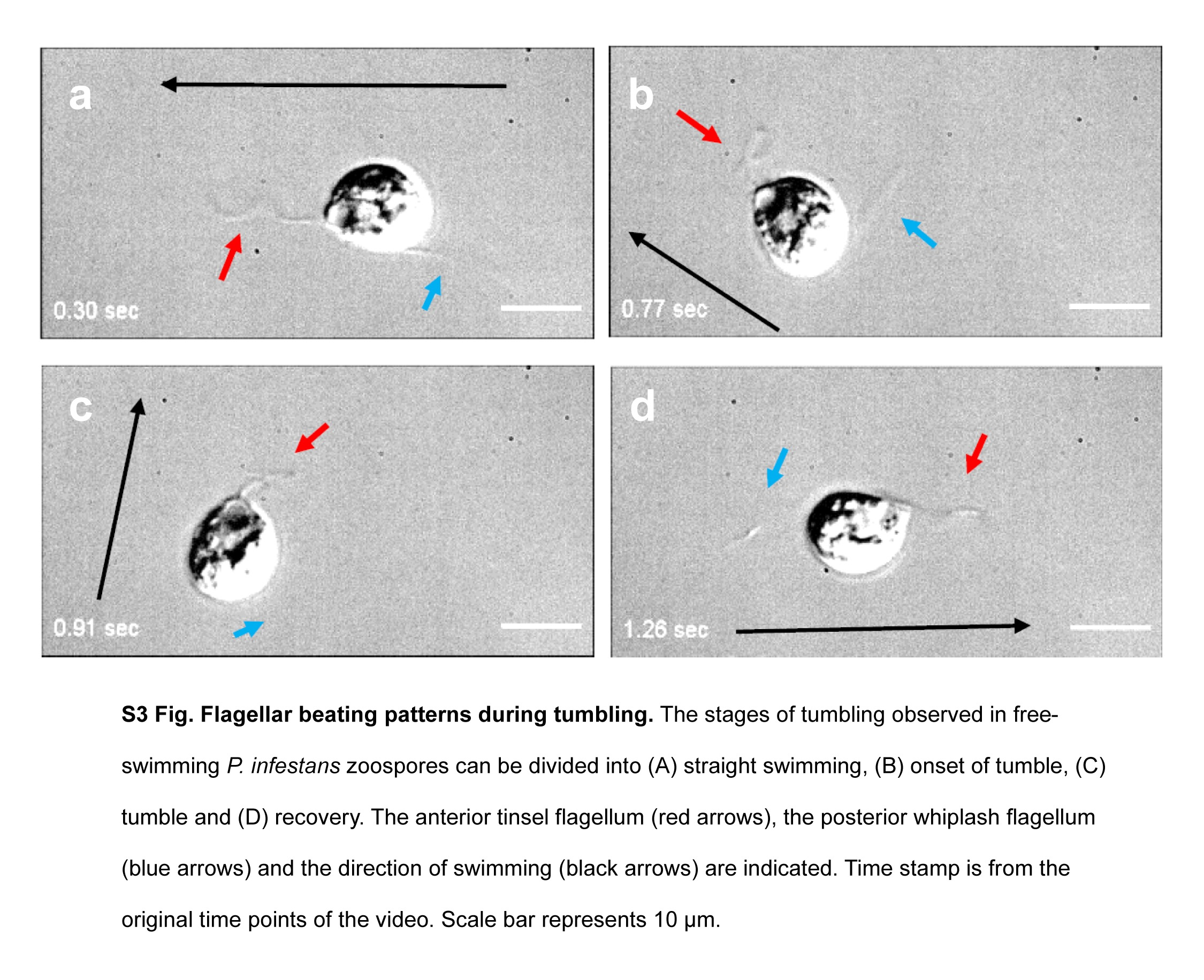

Supplement: S3 Fig — The stages of tumbling observed in free-swimming P. infestans zoospores can be divided into (A) straight swimming, (B) onset of tumble, (C) tumble and (D) recovery. The anterior tinsel flagellum (red arrows), the posterior whiplash flagellum (blue arrows) and the direction of swimming (black arrows) are indicated. Time stamp is from the original time points of the video. Scale bar represents 10 μm. (TIF) [file ppat.1012577.s003.tif]

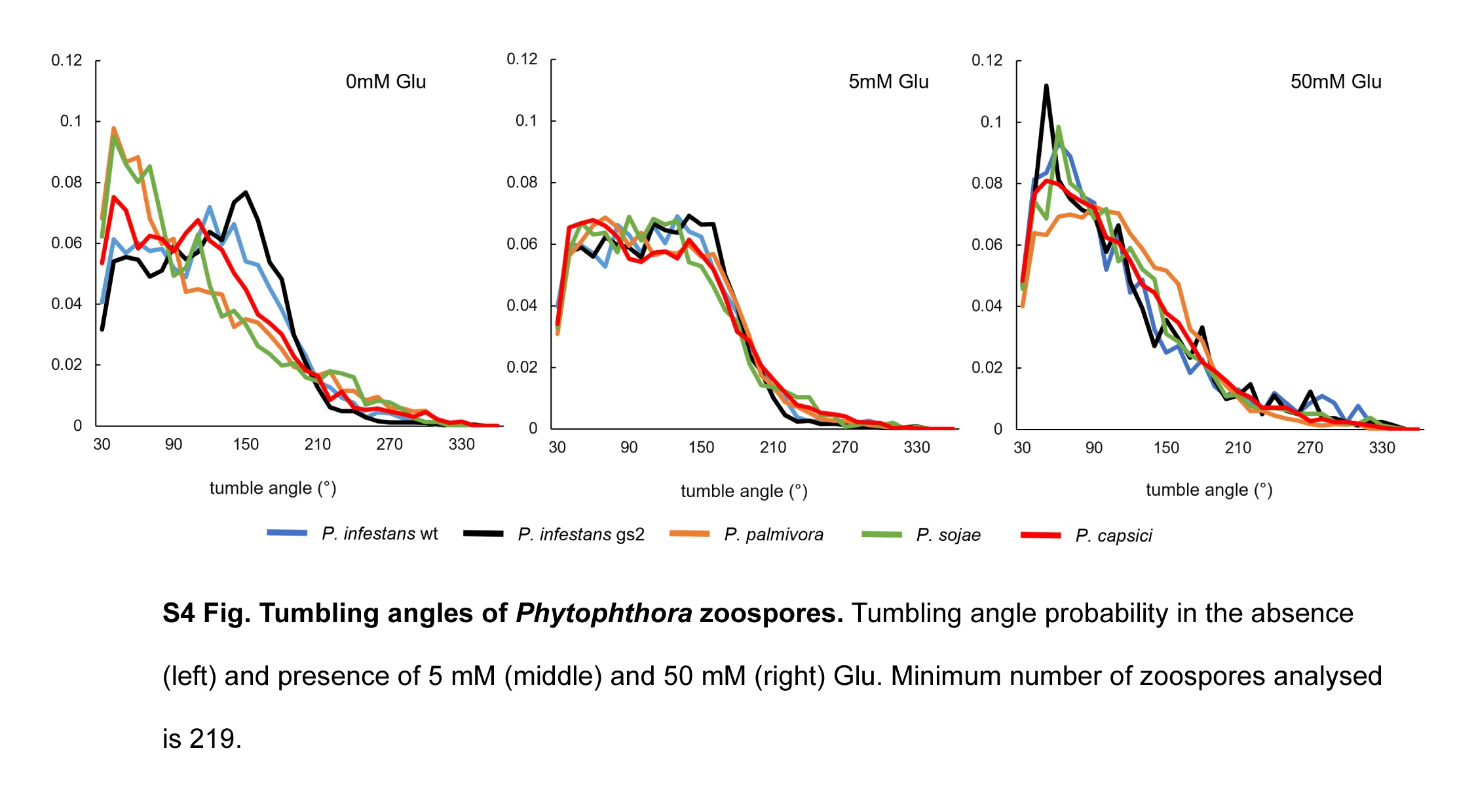

Supplement: S4 Fig — Tumbling angle probability in the absence (left) and presence of 5 mM (middle) and 50 mM (right) Glu. Minimum number of zoospores analysed is 219. (TIF) [file ppat.1012577.s004.tif]

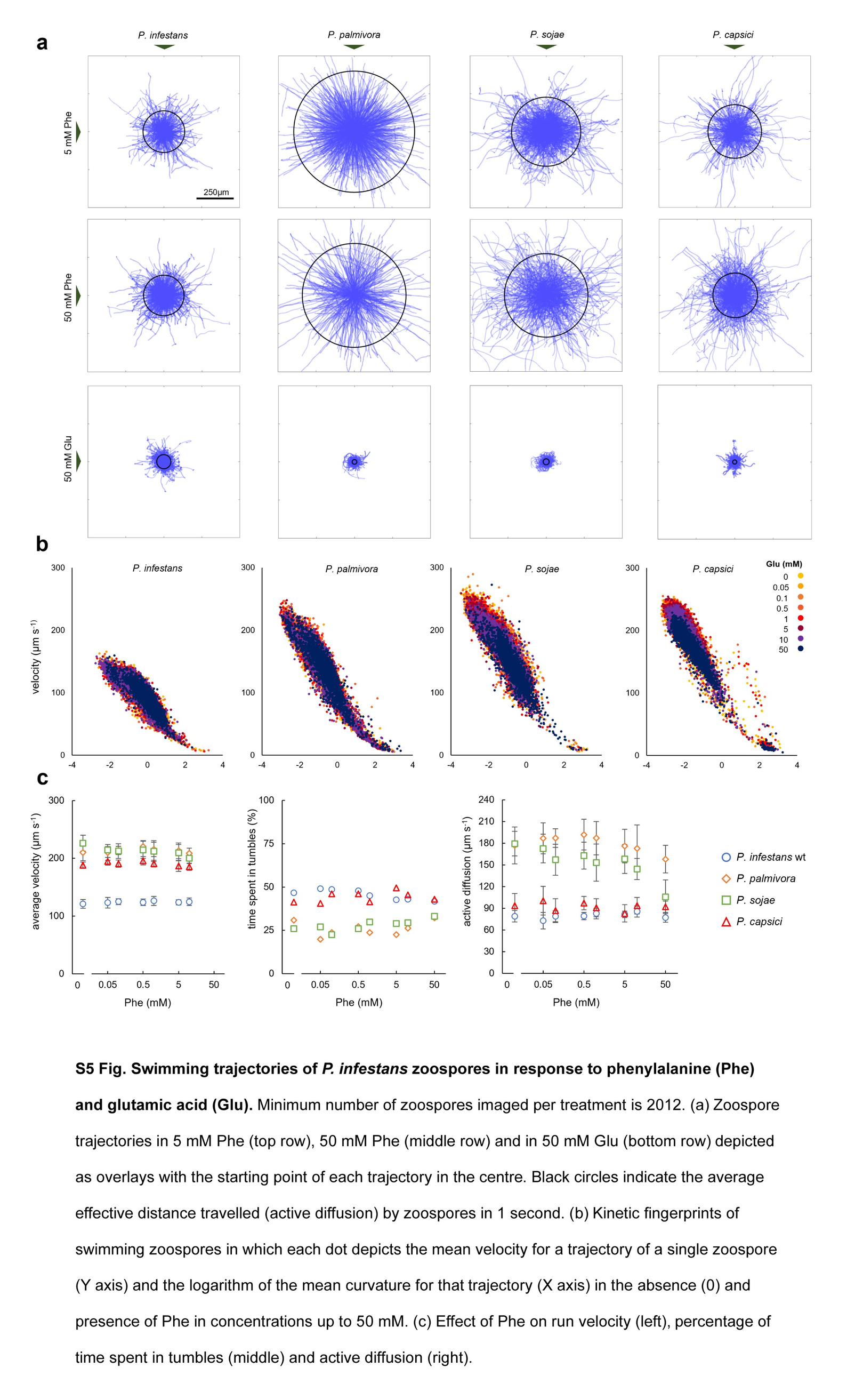

Supplement: S5 Fig — Minimum number of zoospores imaged per treatment is 2012. (a) Zoospore trajectories in 5 mM Phe (top row), 50 mM Phe (middle row) and in 50 mM Glu (bottom row) depicted as overlays with the starting point of each trajectory in the centre. Black circles indicate the average effective distance travelled (active diffusion) by zoospores in 1 second. (b) Kinetic fingerprints of swimming zoospores in which each dot depicts the mean velocity for a trajectory of a single zoospore (Y axis) and the logarithm of the mean curvature for that trajectory (X axis) in the absence (0) and presence of Phe in concentrations up to 50 mM. (c) Effect of Phe on run velocity (left), percentage of time spent in tumbles (middle) and active diffusion (right). (TIF) [file ppat.1012577.s005.tif]

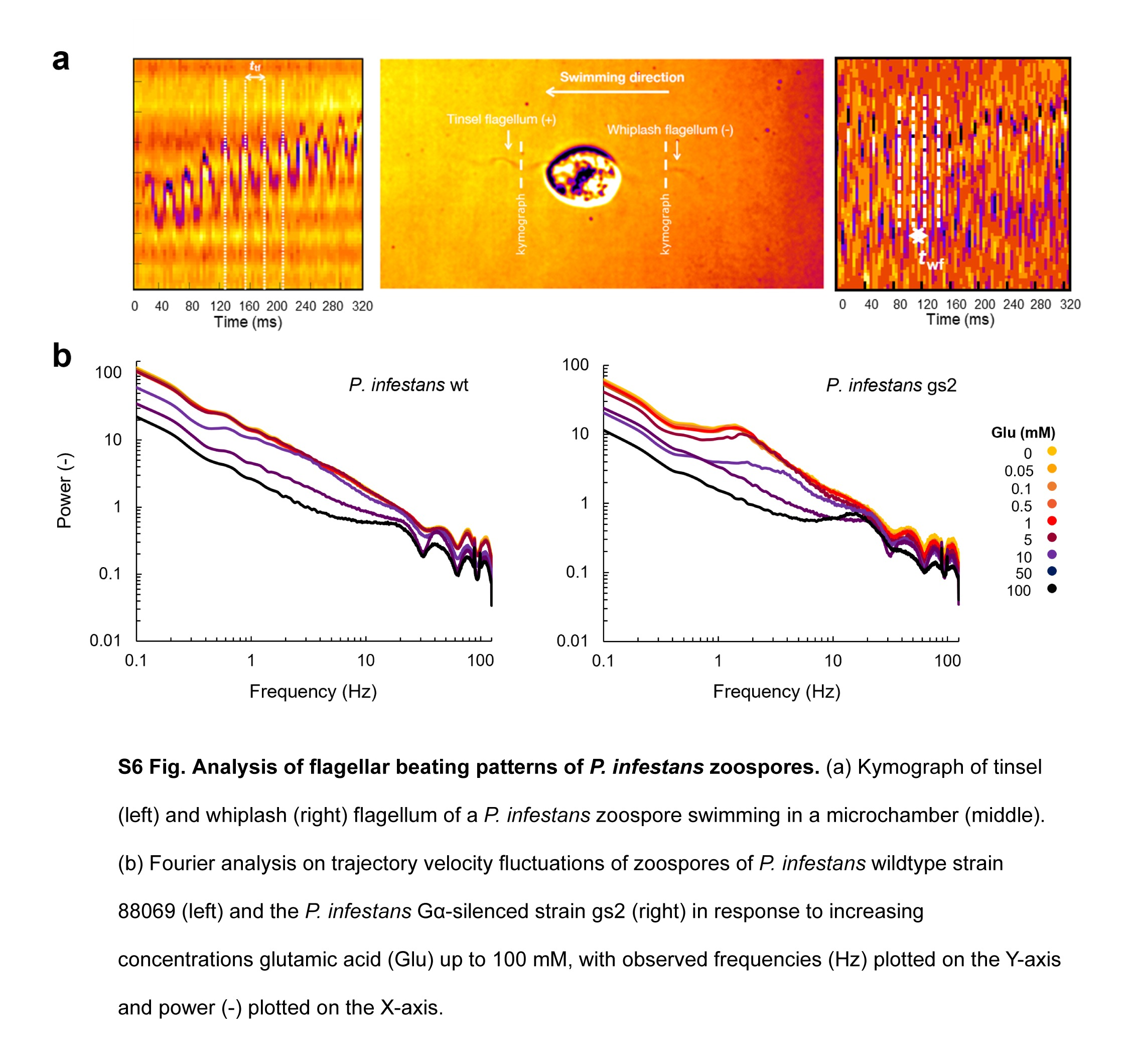

Supplement: S6 Fig — (a) Kymograph of tinsel (left) and whiplash (right) flagellum of a P. infestans zoospore swimming in a microchamber (middle). (b) Fourier analysis on trajectory velocity fluctuations of zoospores of P. infestans wildtype strain 88069 (left) and the P. infestans Gα-silenced strain gs2 (right) in response to increasing concentrations glutamic acid (Glu) up to 100 mM, with observed frequencies (Hz) plotted on the Y-axis and power (-) plotted on the X-axis. (TIF) [file ppat.1012577.s006.tif]

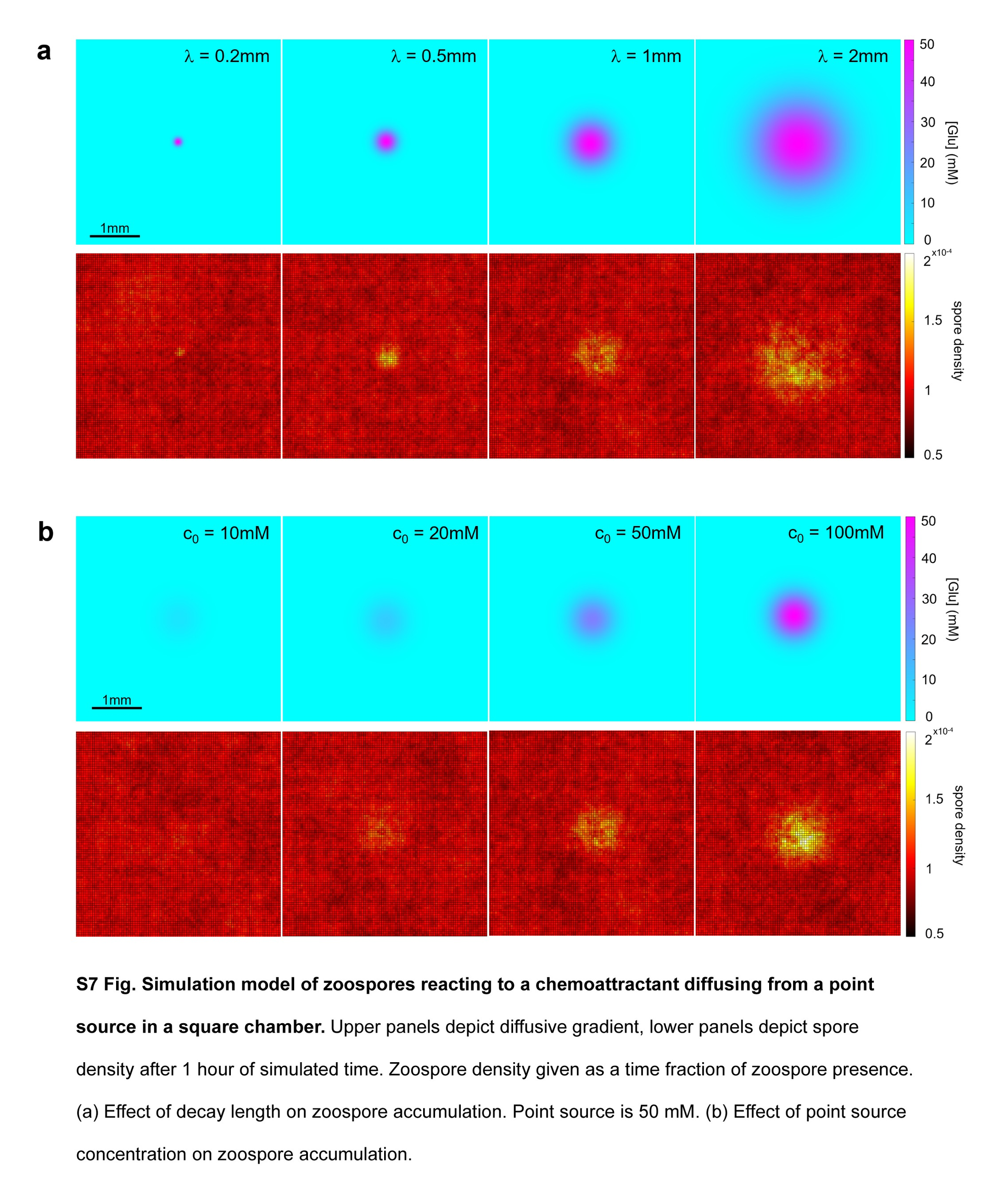

Supplement: S7 Fig — Upper panels depict diffusive gradient, lower panels depict spore density after 1 hour of simulated time. Zoospore density given as a time fraction of zoospore presence. (a) Effect of decay length on zoospore accumulation. Point source is 50 mM. (b) Effect of point source concentration on zoospore accumulation. (TIF) [file ppat.1012577.s007.tif]
