# Supplementary material for: Phytophthora zoospores display klinokinetic behaviour in response to a chemoattractant
Source: PLoS Pathog. 2024 Sep 30;20(9):e1012577. doi: 10.1371/journal.ppat.1012577 (PMC11554144; doi:10.1371/journal.ppat.1012577)
Supplement: S1 Table — (PDF) [file ppat.1012577.s008.pdf]

Supporting information

Belonging to the manuscript entitled '*Phytophthora* zoospores display klinokinetic behaviour in response to a chemoattractant'

AUTHORS: Michiel Kasteel, Tharun P. Rajamuthu, Joris Sprakel, Tijs Ketelaar & Francine Govers

Corresponding author: Tijs Ketelaar, tijs.ketelaar@wur.nl

Table S1. *Phytophthora* strains used in this study.

**Species**	**strain**	**Origin**	**Reference**
P. infestans	88069	Field isolate collected in 1988 in Bennekom, The Netherlands from tomato. A1 mating type.	(1)
P. infestans	gs2	*Pigpa1*-silenced line derived from a DNA transformation of strain 88069.	(2)
P. sojae	P6497	Isolated in Mississippi, USA from soybean.	(3)
P. palmivora	P6390	Field isolate collected in South Sulawesi, Indonesia from coconut. A2 mating type.	(4)
P. capsici	BYA-5	Field isolate collected in 2011 in Gansu Province, China from pepper.	(5)

1. Pieterse CM, Risseeuw EP, Davidse LC. An in planta induced gene of *Phytophthora infestans* codes for ubiquitin. Plant molecular biology. 1991;17:799-811.
2. Latijnhouwers M, Ligterink W, Vleeshouwers VG, Van West P, Govers F. A Gα subunit controls zoospore motility and virulence in the potato late blight pathogen *Phytophthora infestans*. Molecular Microbiology. 2004;51(4):925-36.
3. Förster H, Tyler B, Coffey M. *Phytophthora sojae* races have arisen by clonal evolution and by rare outcrosses. 1994.
4. Mchau GR, Coffey MD. Isozyme diversity in *Phytophthora palmivora*: evidence for a southeast Asian centre of origin. Mycological Research. 1994;98(9):1035-43.
5. Miao J, Cai M, Dong X, Liu L, Lin D, Zhang C, et al. Resistance assessment for oxathiapiprolin in *Phytophthora capsici* and the detection of a point mutation (G769W) in PcORP1 that confers resistance. Front Microbiol. 2016;7:191638.
