# Supplementary material for: Phytophthora zoospores display klinokinetic behaviour in response to a chemoattractant
Source: PLoS Pathog. 2024 Sep 30;20(9):e1012577. doi: 10.1371/journal.ppat.1012577 (PMC11554144; doi:10.1371/journal.ppat.1012577)
Supplement: Attachment — Submitted filename: Kasteel Cover Letter and Response.pdf [file ppat.1012577.s009.pdf]

Dear Editors,

Thank you very much for handling our manuscript entitled ‘*Phytophthora* zoospores display klinokinetic behaviour in response to a chemoattractant’. We are pleased with the overall positive evaluation and thank the reviewers for their feedback.

As per request, we have modified the manuscript. Please find our point-by-point responses to the issues raised by the reviewers and a description of the changes in the revised version of the manuscript below.

Besides a clean version of the manuscript (uploaded as file ‘Kasteel Main Text’), we include a version of the manuscript (file ‘Kasteel Main Text marked’) highlighting the textual changes that were made.

We hope that this modified version can now be accepted for publication in PLOS Pathogens. We are looking forward to your response.

Very best regards, also on behalf of the other authors,

Michiel Kasteel

Responses to the comments of the reviewers in Part I of the questionnaire

We thank the reviewers for evaluating our manuscription and for their positive and enthusiastic comments. We are pleased that they appreciate our research by stating that this is 'certainly a topic of potential interest to a wider audience' (reviewer#1), that it 'provides valuable insights into the chemotactic responses of zoospores of the genus *Phytophthora'* (reviewer#2), and that it is 'suitable for publication in PLOS Pathogens' (reviewer#3).

Responses to issues raised in Part II of the questionnaire by reviewer #1. Reviewer #2 and #3 had no concerns.

The present study does not take into account some very old literature about the role of calcium and its affect on the frequency of turning. I surmise that a calcium influx mediated via a G coupled receptor almost certainly provides the central intracellular signal to coordinate the same response from different types of chemoreceptors.

1. Reid, B., Morris, B. W., and Gow, N. A. R. 1995. Calcium-dependent, genus specific, autoaggregation of zoospores of phytopathogenic fungi. Exp. Mycol. 19: 202–213.
2. Donaldson, S. P., and Deacon, J.W. 1993. Changes in motility of Pythium zoospores induced by calcium and calcium-modulating drugs. Mycol. Res. 97: 877–883.
3. Connolly MS, Williams N, Heckman CA, Morris PF. Soybean isoflavones trigger a calcium influx in Phytophthora sojae. Fungal Genet Biol. 1999 Oct;28(1):6-11. doi: 10.1006/fgbi.1999.1148. PMID: 10512667.
4. Xu, C., & Morris, P. F. (1998). External calcium controls the developmental strategy of Phytophthora sojae cysts. Mycologia, 90(2), 269–275. https://doi.org/10.1080/00275514.1998.12026906

We thank reviewer#1 for pointing out 'old literature' that might be relevant for the discussion. For convenience we have numbered the suggested references. We are aware that there are quite some publications, mainly from the previous century, deal with the role of calcium in oomycete biology. Calcium plays a role in numerous cellular processes in eukaryotes, also in oomycetes. We are familiar with the references mentioned above but the comments of reviewer#1 stimulated us to revisit these publications and other potential sources we may have missed. We want to emphasize that in this manuscript, we focus on just one life stage of *Phytophthora,* i.e., zoospores, and more specifically on zoospore swimming behaviour. We therefore choose to limit the discussion to aspects related to swimming behaviour. In one of the discussion paragraphs, we consider the role of calcium. Here we refer to reference 2 by Donaldson & Deacon (1993) as they indeed describe experiments testing the role of calcium availability on the swimming behaviour of zoospores, specifically those of the oomycete *Pythium aphanidermatum*. To our knowledge, there are no other reports describing the direct role of calcium on swimming patterns or flagellar function in oomycetes. We have revisited this paragraph and have included additional references where appropriate. The modified paragraph is as follows:

> The regularity (Fig S6) and increase (Fig 4d) of tumbling of zoospores of the Gα-deficient *P. infestans* mutant hints to involvement of Gα in tumble suppression. We show that tumbling is the result of flagellar desynchronization (Fig S3) in line with observations on *P. parasitica* zoospores (30). For other microswimmers, such as *C. reinhardtii* and sperm cells, it has been shown that flagellar beating is governed by calcium fluxes (51-53), and that reorientation of the flagella is correlated with increased calcium influx rates (54). As in other eukaryotes, calcium is a key second messenger in oomycetes (55). There are clues that show calcium to play a role in zoospore chemotaxis and autotaxis (28), and calcium fluxes are associated with encystment (56) and the perception of chemoattractants (57). Moreover, manipulation of extracellular and internal calcium levels by calcium modulators has shown that calcium plays a role in controlling swimming patterns, with different modulators invoking distinct changes such as perpetual circular or straight swimming, slow spiral 'skidding', or irregular jerky movements (26). The tumbling increase we observe in the Gα-deficient mutant could well be the result of disturbance of calcium homeostasis, with regular influxes of calcium inducing consistent tumbling. Notability, analyses of *P. sojae* Gα-deficient mutants pointed to Gα as potential negative regulator of intermediates in calcium signalling such as calmodulin and calmodulin-dependent protein kinases (21). In general evidence for involvement of G-protein signalling components in maintaining calcium oscillations is accumulating (58, 59) but to what extent they influence flagellar beating patterns in oomycetes in unknown. Thus, beyond roles in chemoreception, it is worthwhile to investigate the interplay between G-protein mediated signalling and calcium oscillations and influx, and how this affects flagellar beating.

I too have noted that 1mM Ca promotes very straight swimming in response to a chemotactic signal.

We were not able to find publications describing or confirming the observation of reviewer#1 that '1mM Ca promotes very straight swimming in response to a chemotactic signal'. It is not clear to us how we should deal with this remark. It certainly shows the enthusiasm of reviewer#1 for the topic and this interesting observation indeed warrants further investigation into the role of calcium in the homing response. However, this was not the aim of the current study and therefore beyond the scope of this manuscript. For these reasons we choose to take this remark for granted. We have

not incorporated (discussions related to) this remark in the revised manuscript. We hope that the editors appreciate our choice.

Responses to issues raised in Part II of the questionnaire by reviewer #2. Reviewer #1 and #3 had no concerns.

Could the authors provide a rationale for not including L-Phenylalanine as a control in the experiment analysing zoospore movement of P. infestans gs2? The inclusion of L-phenylalanine as an additional control could help to further validate that the ability of gs2 to respond to chemotaxis was not affected.

Phenylalanine is an amino acid that has no activity as attractant for *Phytophthora* zoospores. This has been reported in several publications and we have confirmed this by showing in Fig S5 that the wildtype *P. infestans* isolate 88069 does not adapt its swimming behaviour in response to L-Phenylalanine. It is very unlikely that the Pigpa1-silenced line gs2, which is a derivative of isolate 88069 (Table S1), would show a divergent response to L-Phenylalanine. For addressing the role of G-protein mediated signalling in swimming behaviour and perception of glutamic acid, the inclusion of L-phenylalanine as an additional control has no added value.

In the Results section, lines 153 and 174-177, the manuscript refers to figures as Fig. S2a, b, c. However, the sub-figures do not have the corresponding letter annotations in Fig. S2, and there is no description of the letter annotations in the figure legend.

Apologies for this confusion. In the revised manuscript we removed the letter annotations when referring to Figure S2.

On lines 301 and 303, the manuscript refers to Fig. 3d, but this figure is not included in the submitted material. Could this be a case of mislabelling, and did you intend to refer to Fig. 4d instead?

Thank you for noticing this. Indeed, this is a case of mislabelling that has now been corrected.

Line 270, the second half of the sentence beginning with "and why" seems disjointed and unclear in its current form. It would benefit from rephrasing to ensure that the reason for oomycete zoospores' tendency to tumble upon collision with a surface is explained in a way that is logically connected to the first part of the sentence.

We have rephrased the sentence as follows: *Tumbling to prevent getting stuck might also allow oomycete zoospores to successfully migrate through the complex geometry of the soil (44-46), as exemplified by a zoospore’s tendency to tumble upon collision with a surface (29).*
